# Tissue Distribution, Pharmacokinetics, and Effect of Hematological and Biochemical Parameters of Acute Intravenous Administration of Silver Nanoparticles in Rats

**DOI:** 10.3390/nano14010029

**Published:** 2023-12-21

**Authors:** Elsayed I. Salim, Khaled Y. Abdel-Halim, Mostafa E. El-Mahalawy, Haitham A. Badr, Hafiz Ahmed

**Affiliations:** 1 Research Laboratory of Molecular Carcinogenesis, Department of Zoology, Faculty of Science, Tanta University, Tanta 31527, Egypt; elsayed.salim@science.tanta.edu.eg (E.I.S.); melmehlawy@yahoo.com (M.E.E.-M.); 2 Mammalian & Aquatic Toxicology Department, Central Agricultural Pesticides Laboratory (CAPL), Agricultural Research Center (ARC), Dokki, Giza 12618, Egypt; khaled_yassen68@yahoo.com; 3 Biochemistry Department, Faculty of Agriculture, Zagazig University, Zagazig 44519, Egypt; egy.hab@gmail.com; 4 GlycoMantra Inc., 1450 South Rolling Road, Baltimore, MD 21227, USA

**Keywords:** silver nanoparticles, pharmacokinetics, tissue distribution, hematological and biochemical alterations, rats

## Abstract

The widespread biomedical and commercial applications of silver nanoparticles (AgNPs) have increased their potential for human and environmental exposure and toxicity to human health. The bio-distribution and toxicity of AgNPs in rodents following inhalation, intratracheal instillation, and oral ingestion are well documented; however, little is known about the bio-distribution of intravenously (IV)-administered AgNPs and their organ-specific pathophysiological effects. Here, we investigate the pharmacokinetic pattern and tissue distribution of AgNPs in male rats following IV administration. The animals were humanely sacrificed after 10 min, 1 h, 6 h, 12 h, 24 h, and 168 h of AgNP administration, and the silver (Ag) content was measured from blood samples and various tissues following acid digestion. The AgNPs were readily absorbed and subsequently distributed into most organs predominantly in the colon, small intestine, kidney, and heart after 6 h; however, they were the highest in the spinal cord after 168 h. White blood cells (WBCs) were significantly increased (42–60%) in AgNP-administered animals at all time points except 10 min. Regarding platelets, all AgNP-administered animals showed counts 7.8–39.2% lower, with the lowest count at 168 h post-administration. In the case of lymphocytes (LYMs), the AgNP-treated animals exhibited a count 19.5–41% lower at 10 min and 1 h post-administration; however, the animals at 168 h post-administration showed a count 30.5% more. The mean corpuscular hemoglobin (MCH) counts from the AgNP-treated animals were decreased by 50–62%. The concentrations of aspartate transaminase (AST), urea, and creatinine were increased in the AgNP-treated animals. Taken together, the results suggest that the acute IV administration of AgNPs alters metabolic and hematological parameters in animals and may pose a health risk to humans.

## 1. Introduction

Among several metal nanoparticles (NPs), silver nanoparticles (AgNPs) have been recognized as useful tools in biomedical and commercial applications [1] due to their unique chemical, physical, and biological intrinsic properties, such as a high surface/volume ratio, ease of synthesis, tunable surface chemistry and surface functionalization, and good penetration and traceability in the organism [2]. The commercial and biomedical applications of AgNPs include their use as catalysts and optical receptors in biosensors [3], bio-imagers [4], cosmetics, electronics, and textile engineering; as antimicrobial coatings of medical devices, silver-coated intravenous (IV) catheters in medicinal applications to reduce bacterial infections [5,6,7]; and as orthopedic bone cement, surgical mesh, vascular stents, and wound dressings [8]. Moreover, AgNPs are used as antifungal [4], antiparasitic [9], antiviral [10], and anticancer agents [11] and as drug carriers [12]. These widespread applications, in turn, have increased the potential of AgNPs for human and environmental exposure and toxicity to human health [9]. After their exposure, AgNPs can induce inflammation and oxidative stress at the site of exposure. Moreover, they can cross various biological barriers and enter the systemic circulation. IV-administered AgNPs are directly available in circulation. From there, AgNPs are distributed to multiple organs and cause organ-specific pathophysiological effects.

In vivo bio-distribution and toxicity studies in rodents have demonstrated that AgNPs that are administered by inhalation, ingestion, or IV/IP injection are subsequently detected in blood and cause toxicity in several organs, such as the lungs, liver, kidney, intestine, and brain [4,13]. Inhalation is believed to be a major route of exposure, not only during the manufacturing of Ag-containing materials but also during the use of aerosolized products. The bio-distribution and toxicity of AgNPs in rodents following pulmonary exposure via inhalation and intratracheal instillation are well documented [4].

The presence of AgNPs in dietary supplements, edible fish, and other aquatic organisms provides potential sources of oral exposure [6]. After oral ingestion, AgNPs can act on the mucus layer of the gastrointestinal tract, translocate to the bloodstream, and consequently access each organ upon crossing the epithelium. In epithelial cells, the uptake of NPs with a diameter lower than 100 nm occurs mainly by endocytosis [9]. Within enterocytes, AgNPs trigger oxidative stress, DNA damage, and inflammation [14]. After entering the biological system, nanoparticles may cause biochemical transformation, leading to secondary particles inducing long-term harmful effects on human health [15]. Several studies have reported on the bioavailability and pharmacotoxicity of orally administered AgNPs in various organs. For example, AgNPs exerted developmental and structural malformations in model organisms [16] and histopathologic abnormalities in the spleen, liver, skin, and other organs [17], as well as immunotoxicity [18] and genotoxicity [6,8]. Few studies have investigated the tissue distribution of silver in mammals following different dosage patterns. The oral administration of ionic and nano forms of silver in rats was found to be deposited in a wide range of organs [19], including the glomerular basement membrane [14]. Regarding brain uptake, silver administered through drinking water was localized to the glial cells and neurons of the hippocampus and pons, where its effect was not bound to oxidative stress [20]. On the other hand, several investigations have demonstrated that some organs are more prone to silver deposition than others. For example, Pelkonen et al. found that, following the administration of ionic silver of 0.03 mg/L in drinking water to rats for 1 or 2 weeks, the greatest proportions were found in the soleus muscle, lungs, cerebrum, gastrocnemius muscle, liver, kidneys, and blood [21]. Another investigation by Loeschner et al. documented that the oral administration of a daily dose (9 mg/kg/day) of ionic Ag or AgNPs in rats for 28 days showed different organ distribution [22]. While the highest amount of ionic Ag or AgNPs (35 μg/g tissue) was found in the small intestine, a much lower amount (3 to 10 μg/g tissue) was distributed in the stomach, liver, and kidneys. The lungs, muscles, brain, and plasma received the lowest amount of ionic Ag or AgNPs (less than 1 μg/g tissue). 

In contrast to other routes, IV-administered AgNPs in the form of anti-cancer drugs or as diagnostic modalities are directly available in circulation and are distributed to various organs. However, the bio-distribution of IV-administered AgNPs and their organ-specific pathophysiological effects are not well documented. In the present study, we aim to investigate the pharmacokinetic pattern and tissue distribution of silver nanoparticles in male rats following the acute IV administration of 1 mg/kg body weight, with particular attention given to its terminal half-life (t_0.5_) and triexponential disappearance from the serum and different tissues. 

## 2. Material and Methods

### 2.1. Chemicals

Silver nanoparticle (AgNP) colloids were obtained from Nano Tech. (Dream Land, 6-October City, Egypt) and suspended in 0.9% trisodium citrate. Nitric acid (HNO_3_; 69%), hydrochloric acid (HCl; 30/34%), and hydrogen peroxide (H_2_O_2_; 30%) were supplied by the Central Drug House (P) Ltd. (New Delhi, India). Deionized water was used in all experimental procedures. 

### 2.2. AgNP_s_ Characterization

The absorption optical spectra of the AgNP solution were recorded using an Electronic 21D UV-VIS spectrophotometer. All spectra were recorded in air at room temperature. Pure Ag in an aqueous solution was detected by sampling aliquots (0.2 mL) of colloidal suspensions, then diluted into 2 mL of deionized water, and subsequently measured on a UV-VIS spectrophotometer. The maximum absorption was scanned at wavelengths ranging from 350 to 600 nm.

The silver nanoparticles in sodium citrate solution (0.9%) were filtered using filters coated with carbon, mounted on an electron microscope grid (200 mesh, Veco, Eerbeek, Holland), and visualized under a transmission electron microscope (TEM, JEM-100CX) at the Unit of Faculty of Science (Alexandria University, Alexandria, Egypt). The diameters of the AgNPs were measured at a magnification of 35,000×. Another aliquot was coated on the copper grid to be employed for X-ray electron dispersive analysis (EDA) on an OXFORD Instrument at the Electron Microscope Unit (EMU) at the Faculty of Science (Alexandria University, Egypt). The electron gun was set at 25 Kev to eject electrons from the AgNPs. The emitted energy was visualized as a peak response and recorded as a metal percentage.

### 2.3. Experimental Design

Male Wister rats, weighing an average of 175.0 ± 5 g, were obtained from the Holding Company for Biological Products & Vaccines (VACCERA) (Giza, Egypt). The animals were acclimatized at the Zoology Department, Faculty of Science (Tanta University, Tanta, Egypt) animal facility. The animal experimental protocol was approved by The Institutional Animal Care and Use Committee of the Faculty of Science, Tanta University (Approval #IACUC-SCI-TU-0081, dated 1 August 2018). The animals were fed with a standard rat chow diet and tap water ad libitum.

#### 2.3.1. Pharmacokinetics of AgNPs

A total of 30 rats were subjected to a single IV administration of AgNPs (1 mg/kg) [3] and then sacrificed at the following time intervals: 10 min, 1 h, 6 h, 12 h, 24 h, and 168 h. At each time point, 5 animals were dissected, and blood samples were withdrawn from the dorsal vein. Moreover, selected tissues (spleen, liver, heart, kidneys, spinal cord, muscles, skin, adipose tissues, colon, testes, and small intestines) were excised rapidly, weighed, placed in glass vials, and stored at −20 °C until analysis. For a control, 5 age-matched animals were administered with the vehicle (trisodium citrate solution) and sacrificed at 10 min post-administration.

#### 2.3.2. AgNP Quantification

Silver (Ag) metal was extracted from the blood, tissues, urine, and feces by using the acid digestion process as previously described [23]. Briefly, one wet gram of each sample was digested with 5 mL of HNO_3_ in an ultrasonic water bath until a clear solution was achieved, and then Ag was precipitated by adding 0.5 mL of HCl. Then, 200 μL of H_2_O_2_ was added to improve the digestion process. The mixture was diluted, filtered, and supplemented to 30 mL with deionized H_2_O.

The silver content was measured by inductively coupled plasma optical emission spectroscopy (ICP-OES) using the Optima 7000 DV ICP-OES (PerkinElmer Inc., Waltham, MA, USA) and occasionally with the Agilent 4200 MP-AES microwave plasma model (Central Agricultural Pesticides Laboratory, Giza, Egypt). An autosampler delivered the samples into an instrumental cyclonic spray chamber with a mass flow-controlled nebulizer gas flow at 0.65 L/min. The instrument was operated in a fast-sequential mode and featured a cooled CCD detector. Background and spectral interferences were corrected using Agilent’s MP Expert Software. 

The limit of detection (LOD) of Ag was calculated as double the standard deviation of a series of measurements of a solution against the blank absorbance. Working standards were used, and quality assurance procedures and precautions were carried out to ensure the reliability of the results. The samples were carefully handled to avoid contamination. A recovery experiment was performed by spiking untreated tissues with 100 ng of AgNPs. The fortified samples were processed for analysis as described above. 

### 2.4. Toxicokinetic Analysis

Following their administration, the kinetic analysis of AgNPs in whole blood and tissues was performed as previously described [24]. The terminal half-life of the AgNPs was calculated from the elimination rate constants, which were obtained by linear regression of terminal linear exponential decline in the Ag concentration using the following formula:

$$t_{0.5}=0.693/k_{0.5}$$

The total area under the silver concentration versus the time curve from the serum (AUC_serum_) was calculated by the trapezoidal rule and extrapolated to infinity by using the last data point and respective terminal linear exponential decline. The area under the curve was divided into a few trapezoids. The area of each trapezoid was calculated, and the sum of the areas of all trapezoids yielded an estimate of the actual area under the curve.

The apparent volume of distribution (V_d_) of Ag is mathematically defined as the quotient between the amount of Ag in the body and its serum concentration at zero time. A better method for determining the V_d_ is to use the following relationship:

$$V_{d}=dose/{AUC}_{\infty \cdot \beta }$$

Total body clearance (C_L_) is a concept and parameter analogous to renal clearance. The total body clearance can be determined for a chemical whose kinetics can be described by a single or multi-compartment model by the following equation:

$$C_{L}=dose/{AUC}_{0\to \infty }$$

The extent to which AgNPs are absorbed (F) can be obtained by comparison of the area under the serum versus the time curve after oral and IV administration.

$$fractionabsorbed\left(F\right)=\frac{{AUC}_{oral}}{{AUC}_{i.v}}$$

### 2.5. Hematological Quantification

In this step, 1 mL of blood from the experimental animals was collected in a sterilized tube with 0.5 µL of EDTA as an anticoagulant and kept on ice until use. The samples were subjected to a cell counter instrument for complete blood picture (CBC) analysis (Medonic serial no.14641-Swdish).

### 2.6. Biochemical Quantifications

Then, 2 mL of blood was collected in sterilized tubes without anticoagulant for liver and kidney function analysis. The samples were centrifuged at 3000 rpm for 5 min. The supernatant was used to determine the alanine aminotransferase (ALT), aspartate aminotransferase (AST), total bilirubin, and albumin by using colorimetric reagent kits (Bio Med, Hannover, Germany). The final products were subjected to a spectrophotometer (SPEKOL11, Carl Zeiss, Jena, Germany) at 546, 578, and 623 nm for AST, ALT, bilirubin, and albumin, respectively. On the other hand, the urea and creatinine concentrations were measured using colorimetric kits (Bio Med) on a spectrophotometer (SPEKOL11) at wavelengths of 492 and 578 nm. The activities of ALT and AST were expressed as IU/L, while the concentrations of bilirubin and albumin were expressed as mg/dL and g/dL, respectively.

### 2.7. Statistical Analysis

All data were presented as the mean values ±SEM and subjected to analysis of variance (ANOVA), followed by Duncan’s new multiple range test to compare the different groups. In addition, the means of the kinetic analysis and the terminal half-life of the AgNPs were compared for significance by the least significant difference (LSD) at 0.05 levels. The statistical analysis was performed using the CoStat program version 6.451 (Cohort Software Inc., Irvine, CA, USA).

## 3. Results

### 3.1. AgNP Characterization

**Figure 1:**
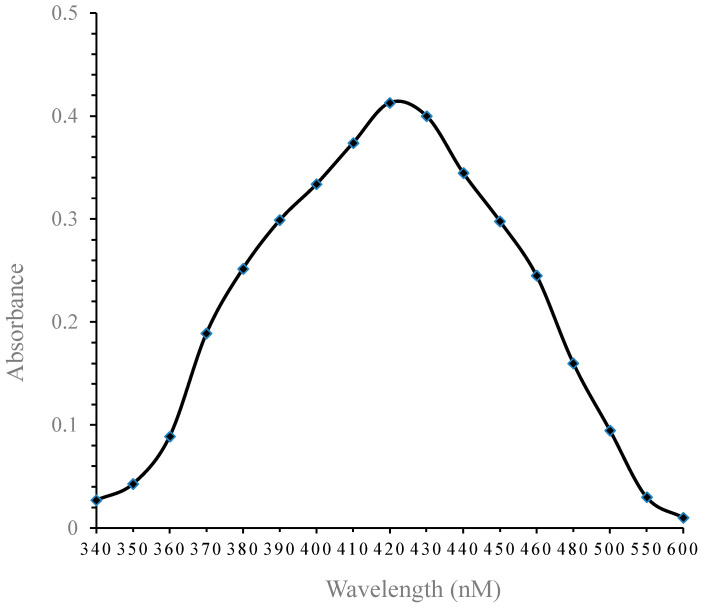
Characterization of AgNPs: UV-VIS absorbance curve of AgNPs, with the absorbance range of 350 to 600 nm indicating silver. The standard blank was deionized water.

The scanned absorbance of UV-VIS ranges for the prepared AgNPs are illustrated in Figure 1. The maximum absorbance was expressed at λmax (420 nm). 

**Figure 2:**
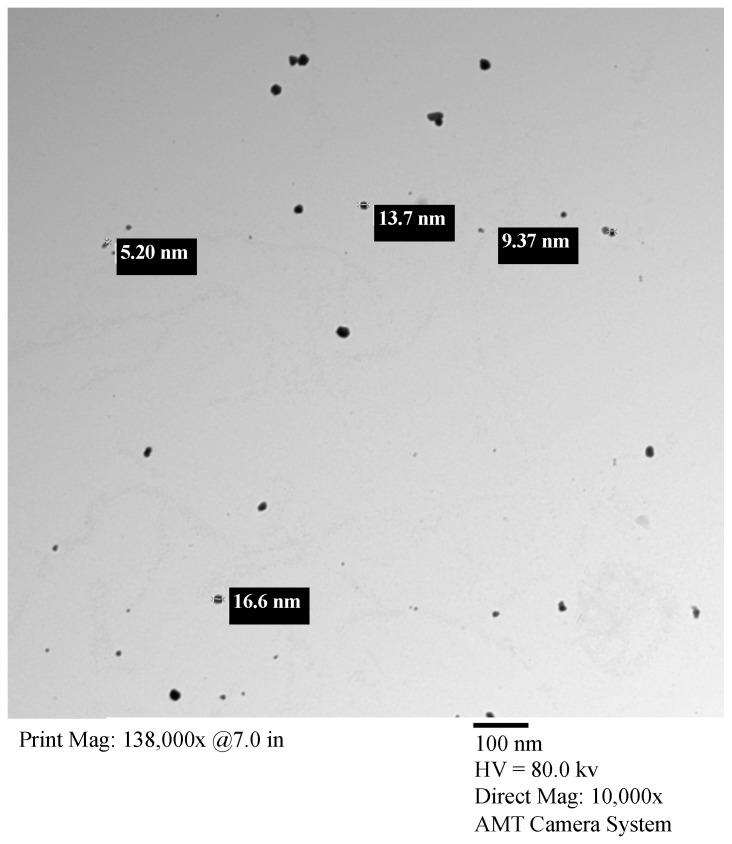
Characterization of AgNPs: transmission electron microscopy (TEM) images of AgNPs are visualized on scales of 5000× and 35,000×. The particle sizes ranged from 4.3 to 16.9 nm.

The AgNPs were nearly spherical particles, with diameters ranging from 4.0 to 18.0 nm, as demonstrated by the TEM images (Figure 2). 

**Figure 3:**
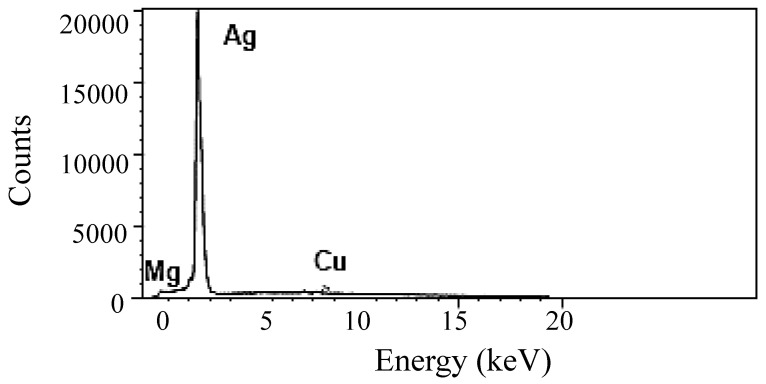
Characterization of AgNPs: electron dispersive analysis (EDA) of Ag set at 25 kev.

According to electron dispersive analysis (EDA), the output of elemental energy exhibited by Ag was >96.6%, while the other metals, such as Mg, Cu and Zn, showed about 3.4% (Figure 3).

### 3.2. Percentage Recoveries and Detection Limits of AgNPs from Tissues and Excreta

Silver nanoparticles (AgNPs) were extracted from the blood, tissues, urine, and feces, as described in Section 2. The recoveries of the AgNPs from the desirable matrix are presented in Table 1. The recovery was highest in the blood and the lowest in the feces. The limit of detection for anionic Ag was set to 0.5 ng/mL.

### 3.3. Tissue Distribution of AgNPs

**Figure 4:**
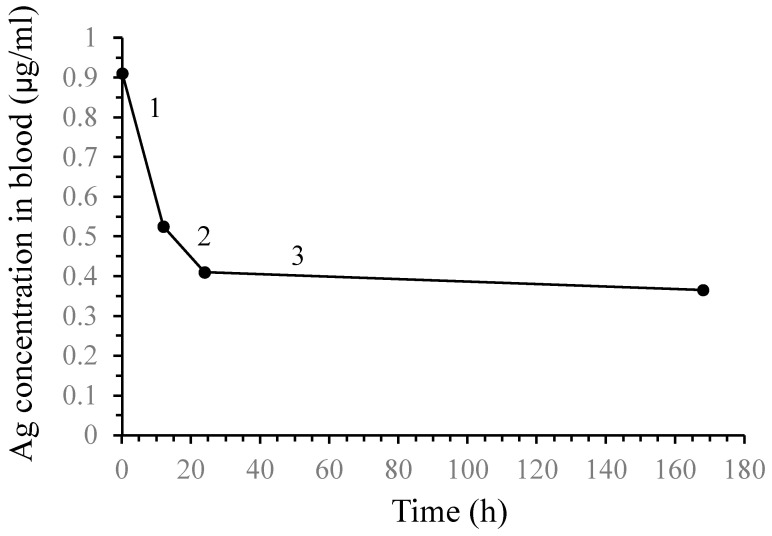
Plot of the concentration of AgNPs in the blood as a function of time after dosing if the body acts as a three-compartment system and the elimination of AgNPs obeys first-order kinetics with a rate constant (K_el_).

The male rats given a single IV dose of AgNPs (1 mg/kg) showed no observable signs of acute toxicity. The decline line of the Ag concentration in the rats’ blood is illustrated in Figure 4. The greatest proportion (0.91 µg/mL) was observed at 10 min, followed by 0.52 µg/mL after 12 h. No significant difference was observed at the other time intervals. 

Regarding the tissue distribution, the AgNPs were readily absorbed and subsequently distributed into the body, as presented in Table 2. Immediately after IV administration, Ag was observed in all analyzed samples. At 10 min post-administration, the metal exhibited the highest proportion in the muscle, followed by the colon, heart, spleen, kidney, and spinal cord. Within 1 h, Ag exerted the highest proportion in the heart and kidney, followed by the spleen and colon. The muscle, testes, and spinal cord also exhibited some Ag (0.132 μg/g), but the small intestine retained the minimum (0.066 μg/g). The colon showed the highest proportions of Ag after 6 h of administration, while the spleen had the highest amount after 12 h of administration. At 168 h post-administration, the spinal cord exhibited the highest amount, and the skin showed the second highest amount.

### 3.4. Excretion of AgNPs

**Figure 5:**
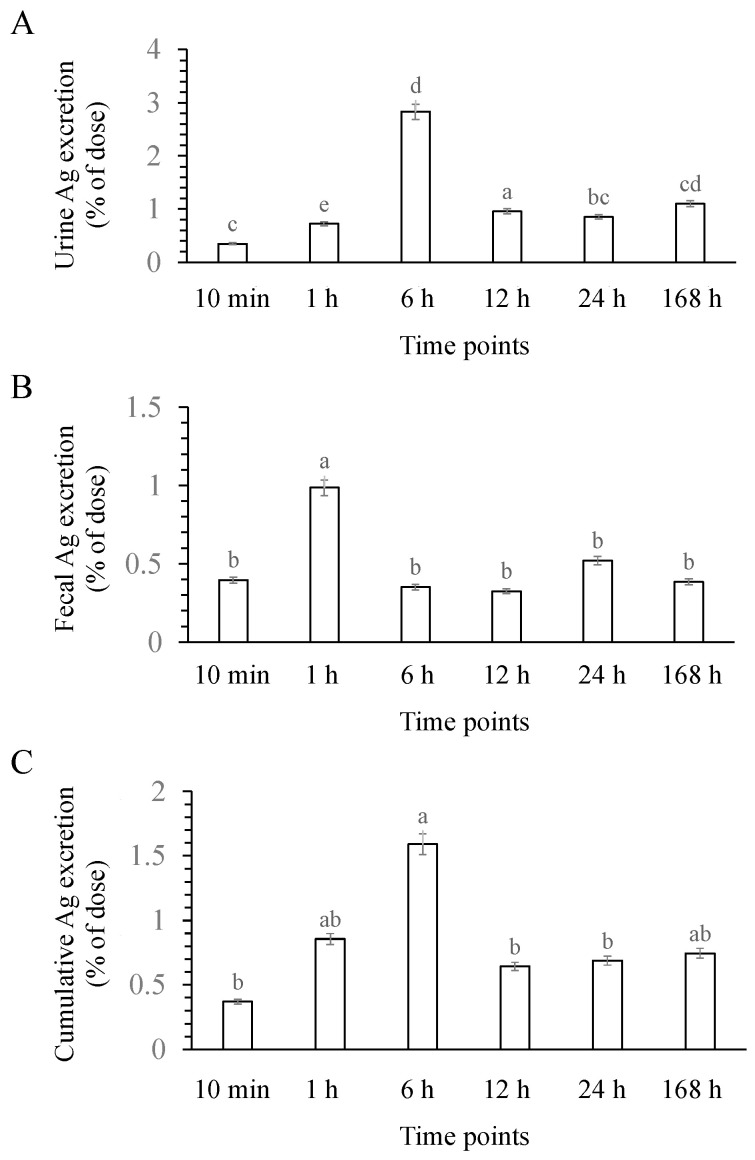
Excretion (% of the applied dose) of AgNPs in male rats after a single IV injection: (**A**) urine, (**B**) feces, and (**C**) cumulative excretion. Each value is the mean of three replicates ± SEM. One-way ANOVA and Duncan’s multiple range test, *p* < 0.05: (**A**), ^a^ vs. all the other time points, ^c^ vs. all the other time points; ^d^ vs. all the other time points; ^e^ vs. all the other time points; ^bc^ vs. all the other time points; ^cd^ vs. all the other time points (**B**) ^a^ vs. all the other time points, ^b^ vs. 1 h; (**C**), ^a^ vs. all the other time points, ^b^ vs. 1, 6, and 168 h, ^ab^ vs. 10 min, 6, 12, and 24 h.

The excretion concentrations of Ag in both the urine and feces are illustrated in Figure 5 (showing the percentage concentration) and Table 3 (showing the amount). It was observed that the excretion in urine was greater than that in feces. In the case of urine, the excretion concentration of Ag exhibited the greatest proportion after 6 h of administration, while the lowest proportion was exerted after 10 min. Among the feces samples, the greatest proportion (1.157 μg/g) was exerted after 1 h, with an excretion percentage of 0.985, while the lowest proportion was 0.382 μg/g after 12 h, accounting for 0.325% of the applied dose. The cumulative excretion percentages were found in the following order: 1.590, 0.855, 0.745, 0.688, 0.643 and 0.373% after 6, 1, 168, 24, 12 h, and 10 min, respectively.

### 3.5. Pharmacokinetics of AgNPs

The concentrations of AgNPs in the whole blood and others as a function of time were determined, while the pharmacokinetic profiles were fitted to triexponential curves according to a three-compartment open-model system. The pharmacokinetic parameters are described by the exponential equation:

$$C_{t}=Pe^{-\gamma t}*A_{{}^{\circ}}e^{-\alpha t}+B_{{}^{\circ}}e^{-\beta t}$$

where C_t_ is the Ag concentration in the organ tissue at time t (h), and α, β, and γ are the rate constants for the first, second, and third phases of the triphasic model, respectively. The pharmacokinetic parameters for Ag in different organs are listed in Table 4. 

In the case of whole blood, the elimination constant (β) was adjusted according to C_°,_ with a value of 0.77 µg/mL, but the other absorption constants β and γ recorded 0.924 and 0.00078 h^−1^, respectively. The terminal half-lives t_0.5α, β,_ and _γ_ were estimated to be 0.1, 0.75, and 8.91 h, respectively. The individual rate constant in compartment 1 (central), K10, was 0.073 h^−1^, while the other constants in compartment 2 (deep), K12 and K21, were 12.09 and 13.71 h^−1^. On the other hand, constants K13 and K31 in the peripheral compartment were 2.77 and 12.03 h^−1^. The ratio of K21/K12 following AgNP administration was found to be 1.06, while that of K31/K13 was 4.34. The area under the curve (AUC_0→∞_) was 64.88 µg/kg/h. Regarding the percentage of bioavailability, the value recorded was 1.81 mL/kg/h, with an absorbed fraction of 1.57. The apparent volume of distribution (V_d_) of metal is mathematically defined as the quotient between the amount of metal in the body and its serum concentration at the zero time point because the metal concentration declines due to elimination and distribution to tissues. The value was 2.009 mL/kg. Finally, the total body clearance (C_L_) is a concept, and the parameter is analogous to renal clearance. 

The pharmacokinetic parameters were estimated in some shallow organs, such as the liver, kidneys, spleen, and peripheral blood, and deep organs, such as the spinal cord, as presented in Table 4. Regarding the liver, the terminal half-lives t_0.5α, β,_ and _γ_ were 0.05, 0.10, and 0.16 h, respectively. The elimination rate constant (K10) was 0.119 h^−1^, while the other constants, K12 and K21, were 787.5 and 808.17 h^−1^, respectively. Constants K31 and K13 were 817.72 and 385.83 h^−1^, respectively. The recorded values for V_d_ and C_L_ were 0.42 mL/kg and 2.91 mL/kg/h, respectively. However, the kidney exhibited a constant (K10) of 0.025 h^−1^, and the other rate constants were lower than those of the liver organ. Moreover, the observed value of V_d_ was 1.35 mL/kg, while the C_L_ value was 2.34 mL/kg/h. The spleen organ exhibited terminal half-lives; the t_0.5α, β,_ and _γ_ were 0.295, 0.43, and 0.59 h, respectively. On the other hand, the constant (K10) was 8.64 h^−1^. The other rate constants, K12, K21, K31, and K13 were 5.12, 0.435, 9.28, and 14.4 h^−1^, respectively. The V_d_ value was 0.85 mL/kg, while the C_L_ value was 1.37 mL/kg/h. The spinal cord exhibited terminal half-lives of the t_0.5α, β,_ and _γ_ lower than those of the other organs. In addition, the V_d_ and C_L_ values were 0.414 mL/kg and 3.185 mL/kg/h, respectively.

### 3.6. Hematological Investigations (Complete Blood Count (CBC))

The data of the CBCs from the AgNP-administered animals are presented in Table 5.

The white blood cell (WBC) counts of the AgNP-administered animals at 1 h to 168 h post-administration were slightly higher compared to the 10 min post-administration of the AgNPs or the vehicle administration. Similar results were also observed for the red blood cell (RBC) counts. Regarding the platelets, all AgNP-administered animals showed 7.8–39.2% decreased counts compared to the control animals, with the lowest count at 168 h post-administration. In the case of lymphocytes (LYMs), the AgNP-treated animals at 10 min and 1 h post-administration exhibited counts 19.5–41% lower; however, the animals at 168 h post-administration showed counts 30.5% higher. Dramatic changes occurred in the mean corpuscular hemoglobin (MCH) from the AgNP-treated animals, which showed 50–62% decreased counts compared to the vehicle-treated control animals. However, the mean corpuscular hemoglobin concentration (MCHC) remained the same in both the AgNP-treated and control animals.

### 3.7. Biochemical Investigations

The results of the biochemical parameters showing the liver and kidney functions of the AgNP-treated animals are presented in Table 6.

Changes in the aspartate transaminase (AST), urea, and creatinine concentrations were evident in the AgNP-treated animals compared to the control animals, while the concentrations of alanine transferase (ALT), total bilirubin, and albumin remained the same in both groups. Notably, the AST, urea, and creatinine concentrations were increased in the AgNP-treated animals, with the highest increase (40–62.5%) at 24 h post-administration.

## 4. Discussion

Pharmacokinetics are quantitative studies of the metabolic processes of adsorption, distribution, metabolism, and excretion (ADME). Thus, the use of different models determines these processes after chemical exposure to living systems. The logarithm of the blood concentration of NPs is considered a function of time that could fit with a single line, suggesting the rapid distribution of NPs into a three-compartment system, as shown in Figure 5. The elimination half-life of AgNPs in serum (0.75 h^−1^) indicates a vast distribution and elimination of the examined AgNPs. Moreover, the elimination of the half-life times in the studied compartments (deep and shallow) induced 0.1 and 8.91 h^−1^. This concept reveals that the AgNPs have a high ability to bind with tissues of shallow organs, e.g., kidneys and spleen, more than deep organs, such as bone marrow, the intestine, and the colon, following IV administration. This finding is similar to the parameters where K10 was 0.073 h^−1^ compared to K21, which accounted for 13.71 h^−1^ (shallow compartment), while K31 accounted for 12.03 h^−1^, and K13 was 2.77 h^−1^ (deep compartment), showing that the blood flow carrying the NPs from the central to shallow compartments was, in one way, a progressive mode.

Moreover, the bioavailability percentage exerted values that were greater than 1, indicating a high ability of the NPs to be distributed among the organs. This is similar to AUC_0→∞_ in serum after IV administration, which exhibited values of 64.88 µg/h/mL. In addition, the volume of distribution (V_d_) exhibited values of 2.009 mL/kg, suggesting that a high blood flow rate increased the distribution of NPs from the central compartment to the organs. Early studies described the systemic deposition of silver using first-order inter-compartment rate constants [13]. Thus, large distractive sampling can lead to a poorly estimated half-lives, and inter-individual variations of Ag concentrations in biological samples can be very high. Thus, the mean residence time (MRT) would seem to better indicate Ag bio-persistence. Similarly, Lee et al. investigated that tissue concentrations of Ag following 28 days of oral administration to Sprague–Dawley rats showed persistence in deep tissues (brain and testes) over 4 months [25]. This finding indicates that the blood–brain and blood–testes barriers play an important role in silver clearance from these tissues. Another investigation demonstrated that AuNPs induced high uptake in the spleen macrophages and Kupffer cells of the liver in rats following IV administration. The uptake was measured by auto-metallography (AMG) [26].

Chemicals are eliminated from the body by various routes. The kidney is a vital organ for their excretion. Toxic compounds are excreted into the urine by the exact mechanisms the kidney uses to remove end products of metabolism from the body. These processes are passive glomerular filtration, passive tubular diffusion, and active tubular secretion. The present finding indicates that the excretion pattern of Ag in urine was greater than that in feces. This concept may be associated with anion characteristics and the route of administration. Furthermore, intravenous administration introduces the chemical directly into the blood stream, and thus, the absorption process is eliminated [27]. The kidney receives about 25% of the cardiac output, and 20% is filtered at the glomeruli. Most chemicals, e.g., NPs, are small enough to be filtered at the glomerulus, and the delivery of kidney-targeted drugs will reduce the undesired side effects of potent drugs [28]. The degree to which a chemical binds to plasma protein affects the filtration rate because a bound chemical is too large to pass through the pores.

On the other hand, chemicals with a high lipid/water partition coefficient will be reabsorbed, and polar compounds and ions will be diffused and excreted into the urine after exposure [10]. Regarding the feces pattern here, the small intestine and colon were established in this mathematical model as deep compartments. Thus, after IV administration the bloodstream slowly transported the AgNP components to them. This concept may explain the low concentrations of NPs in analyzed feces during the experimental period. Moreover, the percentage of bioavailability is over 1, indicating that the absorption and distribution of Ag via IV administration to the urinary tract is greater than those in the digestive system compared to the oral dosage technique.

The proportions of Ag in the liver and kidneys are more than urine-excreted concentrations. This may be explained as follows: the kidney and liver have a high capacity to bind chemicals. These two organs probably have more concentrated toxicants than any other organ. Active transport and protein binding have been suggested as possible mechanisms by which the liver and kidney might remove chemicals from the blood. Recent reports in the literature suggest that the intracellular binding protein may be important in concentrating toxicants in them [29]. In the liver, the Y protein or ligand has been demonstrated to have a high affinity for transferring organic anions from the plasma into the liver [30]. Another binding protein (metallothionein) has been found in the kidney and liver to bind most heavy metals [31]. This phenomenon depends on cysteine-rich protein. For example, Kurasaki et al. reported an induction of Ag-induced metallothionein in the kidney by injecting silver lactate [32]. Another investigation showed that AgNPs induced high bio-persistence in the liver and kidneys after oral administration in rats [23]. In comparison with other routes of AgNPs, the oral dosage of AgNPs was previously shown to be excreted in fecal excretion more than urine, where the Ag content in feces was about 500 times higher than that in urine [7]. This concept may be explained as silver being absorbed from the digestive tract because fecal silver may partially result from biliary excretion. This deserves further investigation.

It is suggested that a wide range of NPs, including AgNPs, may generate reactive oxygen species (ROS) and induce oxidative stress and DNA damage. Their size, surface area, and surface chemistry (e.g., reactive groups) are thought to play a role in the generation of ROS [33]. We have recently reported the induction of 8−hydroxydeoxyguanosine (8−OHdG) adducts in most organs of pregnant rats after the IV administration of AgNPs [6]. This finding implicates that the ADME model of AgNPs in rats shows that the cumulative effect of AgNPs could be toxic to all body organs at certain doses. All the data obtained here indicate that AgNPs can induce risks to exposed organisms by different pathways and route mechanisms within the organism. Thus, these concepts must be considered during NP production and practice programs, especially in food packaging technologies, cosmetics, and medical usage.

AgNPs of varying sizes (2.6–50 nm) have previously been investigated in the cell viability assays of many cancer cells, and the IC_50_ values were determined to be 1.6–7 μg/mL [34]. Our IV dose, 1 mg/kg body weight (means 1 μg/g or 1 μg/mL) was in the lower range of the reported IC_50_ values. Regarding biochemical quantifications, the present data show marked effects of AgNP administration on liver and kidney function after prolonged periods (24 and 168 h). This is in line with what has been shown previously, which is that the concentration of AST was significantly higher in female rats (*p* < 0.05), with a dose of AgNPs 25 nm for 28 days, while the cholesterol (CHOL) and alkaline phosphatase (ALP) concentrations were significantly increased, indicating hepatotoxicity. This was postulated as a relevant marker for AgNP risk to mammals [25]. They also showed that the immune response to montmorillonite/Ag NPs was slightly increased compared to the control animals. This indicates that while not being specifically (cyto)toxic to these cells, exposure to AgNPs has the potential to change the response of immune cells [35]. Collectively, the present findings pointing to the uptake and elimination rates of AgNPs in exposed animals and their localization in some organs, e.g., liver, spleen, and kidney, in addition to correlation with hematological toxicity, indicate that health hazards should be induced in consumers of nanomaterial products.

## 5. Concluding Remarks

As shown in this investigation, IV-administered AgNPs were differentially distributed into various organs; however, these were remarkably stable in the spinal cord, even after 168 h. Changes in the metabolic (AST, urea, and creatinine) and hematological (WBC, platelets, lymphocytes, and MCH) parameters were also evident, suggesting that the IV-administered AgNPs may pose health risks to humans. However, this investigation using five animals per time point is considered a pilot study, and therefore, a further study using a large cohort should be conducted to conclude the use or consumption of AgNPs and the associated health risks.

## Figures and Tables

**Table 1:** Percentage recoveries and detection limits of AgNPs from various tissues and excreta.

Replicates	% Recoveries				LOD (ng/mL)
	Blood	Muscle	Urine	Feces	
R1	100.00	43.88	38.47	78.77	0.5
R2	64.86	46.81	74.17	69.73	
Mean± SEM	82.43 ± 24.84	45.35 ± 2.07	56.32 ± 25.05	74.35 ± 6.39	
LSD (0.05)	17.57	1.46	17.72	4.52	

Two replicates (R1 and R2) were used; LOD, limit of detection; SEM, standard error of the mean; LSD, least significant difference.

**Table 2:** Ag concentrations in various tissues of rat at various time points following a single IV administration of AgNPs.

Time					Ag Concentration (µg/g)						
	Liver	Kidney	Spleen	Heart	Spinal Cord	Adipose Tissue	Colon	Small Intestine	Testes	Muscle	Skin
10 min	0.044 ± 0.95^c^	0.265 ± 0.33^b^	0.265 ± 0.80^b^	0.265 ± 0.33^b^	0.257 ± 3.01^b^	0.044 ± 0.49^d^	0.271 ± 1.03^abc^	0.099 ± 1.67^b^	0.043 ± 1.35^d^	3.384 ± 0.02^a^	0.336 ± 0.66^b^
1 h	0.088 ± 0.48^bc^	0.353 ± 0.25^ab^	0.347 ± 0.67^b^	0.353 ± 0.25^ab^	0.132 ± 5.87^b^	0.088 ± 0.25^c^	0.153 ± 1.83^bc^	0.066 ± 2.51^b^	0.132 ± 0.44^bcd^	0.132 ± 0.49^b^	0.086 ± 2.59^b^
6 h	0.180 ± 0.23^a^	0.441 ± 0.20^a^	0.265 ± 0.87^b^	0.441 ± 0.20^ab^	0.110 ± 7.01^b^	0.088 ± 0.25^c^	0.727 ± 0.38^a^	0.545 ± 0.30^a^	0.286 ± 0.20^a^	0.171 ± 0.37^b^	0.171 ± 1.30^b^
12 h	0.155 ± 0.27^ab^	0.265 ± 0.33^b^	0.731 ± 0.32^a^	0.265 ± 0.33^b^	0.551 ± 1.40^ab^	0.110 ± 0.20^bc^	0.088 ± 3.16^bc^	0.066 ± 2.51^b^	0.198 ± 0.29^ab^	0.176 ± 0.36^b^	0.154 ± 1.45^b^
24 h	0.110 ± 0.38^abc^	0.265 ± 0.33^b^	0.731 ± 0.32^a^	0.265 ± 0.33^b^	0.110 ± 7.01^b^	0.132 ± 0.16^b^	0.080 ± 3.47^c^	0.099 ± 1.68^b^	0.088 ± 0.66^cd^	0.132 ± 0.48^b^	0.154 ± 1.45^b^
168 h	0.176 ± 0.24^a^	0.265 ± 0.33^b^	0.265 ± 0.87^b^	0.265 ± 0.33^b^	1.940 ± 0.40^a^	0.176 ± 0.12^a^	0.595 ± 0.47^ab^	0.132 ± 1.25^b^	0.154 ± 0.38^bc^	0.188 ± 0.34^b^	0.816 ± 0.27^a^
Control	ND	ND	ND	ND	ND	ND	ND	ND	ND	ND	ND

Each value is the mean of three replicates ± SEM. One-way ANOVA and Duncan’s multiple range test, *p* < 0.05: ^a^ vs. all the other time points of all organs (except those with the same superscript symbols); ^b^ vs. 1, 6 h (kidneys, spinal cord), vs. 12, 24 h (spleen), vs. all the other time points (adipose tissue), vs. 6 h (small intestine), vs. 10 min (muscle), vs. 168 h (skin); ^c^ vs. all the other time points of all organs except liver (10 min), adipose tissue (1 and 6 h), colon (24 h); ^d^ vs. all the other time points of all organs except adipose tissue (10 min), testes (10 min); ^ab^ vs. all the other time points (liver, kidney, spinal cord, colon, and testes), vs. 10 min, 12, 24, and 168 h (heart); ^bc^ vs. all the other time points (adipose tissue and testes), vs. 10 min, 6, 24, and 168 h (colon); ^cd^ vs, all the other time points (testes and colon); ^abc^ vs. all the other time points (liver); ^bcd^ vs. all the other time points. ND, not determined.

**Table 3:** Excretion of IV-administered AgNPs in urine and feces at various time points post-administration.

Time After Dosage	Excretion		Total Excretion (µg)
	Urine (µg/mL)	Feces (µg/g)	
10 min	0.410 ± 0.311^c^	0.467 ± 0.633^b^	0.877 ± 0.985^b^
1 h	0.850 ± 0.150^d^	1.157 ± 0.255^a^	2.007 ± 0.351^ab^
6 h	3.315 ± 0.039^a^	0.414 ± 0.714^b^	3.729 ± 0.325^a^
12 h	1.125 ± 0.113^bc^	0.382 ± 0.773^b^	1.507 ± 0.520^b^
24 h	1.015 ± 0.126^cd^	0.607 ± 0.487^b^	1.622 ± 0.385^b^
168 h	1.295 ± 0.099^b^	0.453 ± 0.652^b^	1.748 ± 0.985^ab^

Each value is the mean of three replicates±SEM. One-way ANOVA and Duncan’s multiple range test, *p* < 0.05: ^a^ vs. all the other time points of urine, feces, total excretion except feces (1 h) and total excretion (6 h); ^b^ vs. all the other time points of urine, feces, total excretion except urine (168 h), feces (10 min, 6, 12, 24, and 168 h), and total excretion (10 min, 12, and 24 h); ^c^ vs, all the other time points of urine, feces, total excretion; ^d^ vs, all the other time points of urine, feces, total excretion; ^ab^ vs, all the other time points of urine, feces, total excretion except total excretion (168 h); ^bc^ vs. all the other time points of urine, feces, and total excretion; ^cd^ vs. all the other time points of urine, feces, total excretion.

**Table 4:** Pharmacokinetic parameters of IV-administered AgNP_s_ (1 mg/kg) in blood and various organs of male rat.

Item	Blood	Liver	Kidney	Spleen	Spinal Cord
A_°_ µg/mL or g	0.8	0.04	0.26	0.27	0.27
B_°_ µg/mL or g	0.6	0.005	0.11	0.00	0.19
P µg/mL or g	0.33	0.11	0.73	2.00	0.00
α h^−1^	9.63	13.86	2.31	2.35	3.08
β h^−1^	0.924	6.93	1.73	1.61	7.70
γ h^−1^	0.00078	4.33	2.60	1.17	2.39
t_0.5_ α h	0.10	0.05	0.30	0.295	0.225
t_0.5_ β h	0.75	0.10	0.40	0.43	0.090
t_0.5_ γ h	8.91	0.16	0.37	0.59	0.290
K21 h^−1^	13.71	808.17	161.36	0.435	52.26
K10 h^−1^	0.073	0.119	0.025	8.64	0.45
K12 h^−1^	12.09	787.5	157.35	5.12	41.93
K31 h^−1^	12.03	385.82	12.49	9.28	0.65
K13 h^−1^	2.77	817.75	7.61	14.40	4.37
K21/K12	1.06	1.026	1.03	0.084	1.25
K31/K13	4.34	0.482	1.64	0.644	0.15
Bioavailability%	1.73	-	-	-	-
F	1.57	-	-	-	-
V_d_ ml/kg	2.009	0.42	1.35	0.85	0.414
Cl_b_ ml/kg/h	1.81	2.91	2.34	1.37	3.185
AUC_0→∞_ µg/h/mL or g	64.88	40.43	50.24	85.33	36.83
R_R_	-	0.62	0.774	1.32	0.57

A_°_, extrapolated zero time concentration during absorption phase; B_°_, extrapolated zero time concentration during elimination phase; A, absorption rate constant; β, elimination rate constant; t_0.5_ α, absorption half-life; t_0.5_ β, elimination half-life; K12, first-order rate constant for the passage of compound from central to peripheral compartment; K21, first-order rate constant for the passage of compound from peripheral to central compartment; K10, first-order rate constant of elimination of compound from central compartment; AUC, total area under the curve; V_d_, apparent volume of distribution; CL_b_, total body clearance; K31, first-order rate constant for the passage of compound from central compartment to shallow peripheral compartment; K13, first-order rate constant for the passage of compound from shallow peripheral compartment to central compartment; F, fraction absorbed; bioavailability, comparison of the area under the plasma versus time curve after oral and IV administration; R_R_, relative residence of pesticide (AUC_organ_/AUC_serum_).

**Table 5:** Complete blood count (CBC) levels at various time points post-administration of AgNPs in male rats.

Parameters	Unit	Time Post-Administration						
		10 min	1 h	6 h	12 h	24 h	168 h	Control
WBC	X10³/μL	7.1 ± 1.12^a^	9.8 ± 0.9^b^	9.1 ± 0.92^b^	9.4 ± 0.61^b^	8.5 ± 0.82^b^	8.8 ± 0.71^b^	8.2 ± 0.92^a^
RBC	X10^6^/μL	4.36 ± 0.12^a^	6.5 ± 0.09^b^	7.2 ± 0.05^b^	7.12 ± 0.05^b^	6.8 ± 0.07^b^	6.4 ± 0.03^b^	4.5 ± 0.02^a^
HGB	g/dL	11.2 ± 0.7^a^	11.5 ± 0.9^a^	14.1 ± 0.7^b^	14.3 ± 0.8^b^	14.4 ± 0.7^b^	13.1 ± 0.7^b^	12.5 ± 0.6^a^
HCT	%	46.4 ± 4.2^a^	43.2 ± 4.4^a^	49.4 ± 3.62^a^	45.6 ± 2.5^a^	42.3 ± 2.3^a^	47.5 ± 4.4^a^	45.8 ± 3.3^a^
MCV	Fl	65.6 ± 5.4^a^	73.2 ± 5.5^a^	81.7 ± 7.3^a^	79.2 ± 5.4^a^	78.3 ± 5.3^a^	80.1 ± 5.3^a^	77.1 ± 6.9^a^
MCH	Pg	25.7 ± 1.3^a^	28.2 ± 1.1^b^	23.9 ± 1.1^a^	31.3 ± 1.4^b^	26.8 ± 0.6^a^	27.1 ± 0.7^b^	62.9 ± 0.5^c^
MCHC	g/dL	32.3 ± 2.5^a^	35.1 ± 3.4^a^	35.1 ± 1.2^a^	38.8 ± 5.8^b^	33.1 ± 4.5^a^	38.3 ± 4.1^b^	36.2 ± 1.2^b^
PLT	X10³/μL	320 ± 16.9^a^	277 ± 16.6^b^	215 ± 26.2^b^	230 ± 11.8^b^	253 ± 12.8^b^	211 ± 13.8^b^	347 ± 16.7^a^
LYM	X10³/μL	3.3 ± 0.4^a^	2.4 ± 0.6^b^	4.3 ± 0.4^b^	3.1 ± 0.4^a^	4.3 ± 0.5^b^	5.9 ± 0.1^b^	4.1 ± 0.1^b^
MON	X10³/μL	0.7 ± 0.0^a^	1.3 ± 0.02^b^	1.1 ± 0.01^b^	1.3 ± 0.02^b^	0.85 ± 0.04^b^	0.9 ± 0.04^b^	0.9 ± 0.02^b^
GRA	X10³/μL	3.1 ± 0.04^a^	6.2 ± 0.06^b^	5.3 ± 0.13^b^	7.2 ± 0.04^b^	6.3 ± 0.01^b^	6.9 ± 0.07^b^	5.3 ± 0.3^b^

Each value is the mean of three replicates ± SEM. One-way ANOVA and Duncan’s multiple range test, *p* < 0.05: ^a^ vs. 1, 6, 12, 24, 168 h (WBC, RBC, and PLT), vs. 6, 12, 24, 168 h (HGB), vs. 1, 12, 168 h and control (MCH), vs. 12, 168 h, and control (MCHC), vs. 1, 6, 24, 168 h, and control (LYM), vs. all of the other time points (MON and GRA); ^b^ vs. 10 min and control (WBC, RBC, and PLT), vs. 10 min, 1 h, and control (HGB), vs. all time points and control (HCT and MCV), vs. 10 min, 6, 24 h, and control (MCH), vs. 10 min, 1, 6, 24 h (MCHC), vs. 10 min and 12 h (LYM), vs. 10 min (MON and GRA); ^c^ vs. all of the other time points for all the parameters. WBC, white blood cell; RBC, red blood cells; HGB, hemoglobin; HCT, hematocrit; MCV, mean corpuscular volume; MCH, mean corpuscular hemoglobin; MCHC, mean corpuscular hemoglobin concentration; PLT, platelets; LYM, lymphocytes; MON, monocytes; GRA, granulocytes.

**Table 6:** Biochemical parameter levels in blood samples of IV-administered AgNPs in male rats at various time points post-administration.

Parameters	Unit	Time Post-Administration						Control
		10 min	1 h	6 h	12 h	24 h	168 h	
ALT	IU/L	11.0 ± 0.12	10.0 ± 0.19	12 ± 0.21	11.0 ± 0.13	13.0 ± 0.62^a^	12.0 ± 0.17	11.0 ± 0.21
AST	IU/L	8.0 ± 0.19^a^	9.0 ± 0.19	10 ± 0.15	10.0 ± 0.15	12.0 ± 0.2^b^	11.0 ± 0.11	8.0 ± 0.13^a^
Total bilirubin	mg/dL	0.7 ± 0.07^a^	0.8 ± 0.06	0.8 ± 0.06	0.8 ± 0.03	0.9 ± 0.05	0.9 ± 0.05	0.8 ± 0.04
Albumin	g/dL	4.4 ± 0.14	4.2 ± 0.15	4.4 ± 0.15	4.1 ± 0.14	4.3 ± 0.14	4.1 ± 0.13	4.4 ± 0.13
Urea	(mg/dL)	27.0 ± 7.9	25.0 ± 4.8^a^	31.0 ± 6.3^a^	36.0 ± 6.3^b^	45.0 ± 4.9^b^	43.0 ± 5.1^b^	27.0 ± 7.4
Creatinine	(mg/dL)	0.7 ± 0.03^a^	0.9 ± 0.01	0.9 ± 0.01	1.3 ± 0.04^b^	1.3 ± 0.05^b^	1.3 ± 0.05^b^	0.8 ± 0.03

Each value is the mean of three replicates ± SEM. One-way ANOVA and Duncan’s multiple range test, *p* < 0.05: ^a^ vs. 12, 24, and 168 h (urea and creatinine), vs. 24 h (AST); ^b^ vs. 24 h (ALT), vs. 10 min and control (AST), vs. 10 min (total bilirubin and creatinine), vs. 1 h (urea). ALT, alanine transferase; AST, aspartate transaminase.

## Data Availability

Data are contained within the article.
